# Novel role of PAF1 in attenuating radiosensitivity in cervical cancer by inhibiting IER5 transcription

**DOI:** 10.1186/s13014-020-01580-w

**Published:** 2020-05-29

**Authors:** Jing-Jie Zheng, Yue He, Yang Liu, Feng-Shuang Li, Zhen Cui, Xiao-Meng Du, Chun-Peng Wang, Yu-Mei Wu

**Affiliations:** Department of Gynecologic Oncology, Beijing Obstetrics and Gynecology Hospital, Capital Medical University, Present address: Dong-Cheng District, Qi-He-Lou Street No.17, Beijing, 100006 China

**Keywords:** RNA polymerase II associated factor 1 (PAF1), Immediate-early response 5 (IER5), Enhancer, Apoptosis, Radiosensitivity, Cervical cancer

## Abstract

**Background:**

Radiosensitivity is limited in cervical cancer (CC) patients due to acquired radiation resistance. In our previous studies, we found that immediate-early response 5 (IER5) is upregulated in CC cells upon radiation exposure and decreases cell survival by promoting apoptosis. The details on the transcriptional regulation of radiation-induced IER5 expression are unknown. Studies in recent years have suggested that Pol II-associated factor 1 (PAF1) is a pivotal transcription factor for certain genes “induced” during tumor progression. In this study, we investigated the role of PAF1 in regulating IER5 expression during CC radiotherapy.

**Methods:**

PAF1 expression in CC cells was measured by western blotting, immunohistochemistry, and qRT-PCR, and the localization of PAF1 and IER5 was determined by immunofluorescence. The effect of PAF1 and IER5 knockdown by siRNA in Siha and Hela cells was studied by western blotting, qRT-PCR, CCK-8 assay, and flow cytometry. The physical interaction of PAF1 with the IER5 promoter and enhancers was confirmed using chromatin immunoprecipitation and qPCR with or without enhancers knockout by CRISPR/Cas9.

**Results:**

We confirmed that PAF1 was highly expressed in CC cells and that relatively low expression of IER5 was observed in cells with highly expressed PAF1 in the nucleus. PAF1 knockdown in Siha and Hela cells was associated with increased expression of IER5, reduced cell viability and higher apoptosis rate in response to radiation exposure, while simultaneous PAF1 and IER5 knockdown had little effect on the proportion of apoptotic cells. We also found that PAF1 hindered the transcription of IER5 by promoting Pol II pausing at the promoter-proximal region, which was primarily due to the binding of PAF1 at the enhancers.

**Conclusions:**

PAF1 reduces CC radiosensitivity by inhibiting IER5 transcription, at least in part by regulating its enhancers. PAF1 might be a potential therapeutic target for overcoming radiation resistance in CC patients.

Cervical cancer (CC) is one of the most common malignant tumors of the female reproductive system, with approximately 500,000 new cases diagnosed every year worldwide and more than 200,000 deaths per year [1]. China accounts for 1/3 of new CC cases each year [2]. The mortality of early and mid-stage CC has decreased over recent decades because of improved early diagnosis and surgical therapeutic strategies. However, the prognosis of late-stage CC remains poor, mainly due to the high incidences of tumor recurrence and metastasis. Radiotherapy is the essential treatment for patients with late-stage CC. Given the recent advances in 3D conformal technology, which precisely directs radiation to the cancerous tissue while minimizing damage to surrounding healthy tissue, radiotherapy not only achieves better clinical outcomes but also leads to reduced radiotoxicity. Nevertheless, CC cells acquire radioresistance, which means that they become insensitive to radiation and continue to survive and multiply after radiotherapy, severely limiting the efficacy of radiotherapy and becoming the most critical factor leading to tumor recurrence and metastasis.

Efforts have been made to identify genes responsible for controlling radiosensitivity in CC cells, which involve DNA damage repair [3, 4], cell cycle monitoring [5, 6], apoptosis [7, 8], signal transduction [9, 10], and the tumor microenvironment [11]. Results from our previous study showed that the expression of immediate-early response 5 (IER5) was upregulated in CC tissues after treatment with radiotherapy [12]. IER5, as a member of the early-response protein family, can be stimulated by sleep deprivation, hormones, radiation, drugs, etc. Moreover, the results from our previous study showed that the radiation-induced expression of IER5 enhances radiosensitivity by promoting the apoptosis of tumor cells [13, 14]. Yang, C. et al. [15] found that the negative transcriptional regulation of the IER5 gene promoter region was abolished, resulting in increased IER5 transcription in response to radiation exposure of liver cancer cells. Consistent with this finding, after radiotherapy of colon cancer cells, the binding of RNA polymerase II (Pol II) to the promoter region of the IER5 gene was enhanced, which ultimately caused increased expression of IER5 [16]. These results suggest that the main reason for the upregulation of radiation-induced IER5 may be its increased transcription level. Despite these findings, relatively little is known about the mechanism of upstream transcriptional regulation of the activated IER5 response to the radiation of CC cells.

Human RNA polymerase II-associated factor 1 (hPAF1) complex (PAF1C), composed of six proteins (PAF1, CDC73, CTR9, RTF1, LEO1 and SKI8), is universally conserved across different species [17] and has specific roles in transcriptional elongation, mRNA maturation and processing, histone covalent modification, and telomere silencing [18]. PAF1 is involved in the Pol II-associated network and plays important roles in chromatin organization, oncogenic transformation, stem cell self-renewal and the cell cycle [18]. PAF1 is highly expressed in various cancers, such as pancreatic cancer [19] and lung cancer [20], and is associated with tumor metastasis, minimal differentiation [21], therapeutic resistance [22], and poor prognosis [20]. Overall, these studies validated the positioning of PAF1 at the crossroads of the cancer network [18]. To our knowledge, the role of PAF1 in CC is unknown.

Chen et al. [23] found that in HCT116 cells, knocking down PAF1 triggered the release of Pol II that had paused at the promoter-proximal region of the IER5 gene to resume elongation. However, there is a lack of research on the role of PAF1 in regulating IER5, which is induced by radiation exposure of CC cells, and whether PAF1 is involved in the radiosensitivity of CC cells deserves further study.

In this study, we sought to determine whether PAF1 is abnormally expressed in CC cells and whether PAF1 could regulate IER5 to influence the response of CC cells to radiation intervention. We show, for the first time, that PAF1 is overexpressed in CC cells and might negatively regulate IER5 gene transcription partially by inhibiting enhancers and attenuating radiosensitivity during radiation treatment. These findings provide insights into the development of radioresistance and will help to identify predictors and targets of radiosensitivity in CC cells.

## Materials and methods

### Tissue specimens, cell culture, and radiation exposure

From October 2018 to July 2019, CC tissues were obtained from 15 patients with stage IIB-IIIB CC (FIGO 2018) (median age, 55.4 yrs.; range 43–78 yrs). Healthy cervical tissues were collected from 15 patients (median age, 48.3 yrs.; range 45–60 yrs) undergoing hysterectomy because of uterine myoma. The Beijing Obstetrics and Gynecology Hospital Research Ethics Committee approved this study, and informed written consent was obtained from all patients.

Hela and Siha cells, both with wild-type p53 [24], were grown in DMEM (HyClone) supplemented with 10% fetal bovine serum (Gibco) and antibiotics, namely, penicillin (100 IU/ml) and streptomycin (100 μg/ml), in a humidified incubator at 37 °C with 5% CO_2_. Cells were subjected to irradiation (IR) in our radiology clinic with Co^60^-γ rays at a dose rate of 1.35 Gy/min at room temperature. On the basis of previous studies by our team [25] and other researchers [26], we presumed that 4 Gy is the optimal dose for Hela cells and that 3.5 Gy is the optimal dose for Siha cells.

### Knocking down PAF1 and IER5 with specific siRNAs

Specific siRNAs were purchased from OriGene: CGAGUCAAGUACUGCAAUAGCCUCC (si-PAF1), CCUCAUCAGCAUCUUCGGUUU (si-IER5), and a universal scrambled control siRNA duplex was also obtained from OriGene. Transfection was performed using Lipofectamine 3000 (Invitrogen) following the manufacturer’s recommendations. Cells were harvested 48 h after transfection and subjected to different further assays.

### Western blotting

Frozen tissue samples and Siha and Hela cells were processed for protein extraction and western blotting using standard procedures. In brief, 100 mg of tissue (homogenized first using a homogenizer) and cells were washed twice in phosphate-buffered saline (PBS) and then lysed in radioimmunoprecipitation assay (RIPA) buffer containing 100 mM phenylmethanesulfonyl fluoride (PMSF, Solarbio) and protease inhibitor cocktail. Total protein in the supernatant was quantitated by the bicinchoninic acid (BCA) protein assay kit (BCA protein assay kit, Thermo Scientific). The proteins were resolved by using 8% SDS-PAGE and transferred onto PVDF membranes, which were incubated overnight with primary antibodies (anti-PAF1, 15,441–1-AP, Proteintech; anti-IER5, ab59133, Abcam) at 4 °C. The membranes were then probed with secondary antibodies. Finally, the western blotting bands were exposed using an Amersham Imager 600 (General Electric Company, USA) with an ECL chemiluminescence kit (Millipore Corporation, Billerica, MA, USA). The Western blotting bands were quantified using ImageJ software and normalized to the level of GAPDH.

### RNA isolation and quantitative reverse transcription-polymerase chain reaction (qRT-PCR)

Total cellular RNA was extracted from Siha and Hela cells using TRIzol reagent (Sigma) according to the manufacturer’s instructions. RNA concentration and absorbance were measured by a NanoDrop 1000 spectrophotometer (Thermo Fisher Scientific). cDNA was synthesized using the RevertAid first-strand cDNA synthesis kit (Thermo Fisher Scientific). SYBR Green Real-Time quantitative PCR was performed using SYBR qPCR Mix (Toyobo) with an Agilent Mx3000P system (Stratagene) and analyzed using the comparative C.T. method [27] with the primers shown in Table S1. All the calculated values of the genes were normalized to that of the internal control gene β-actin.

### Immunohistochemistry (IHC)

IHC analysis was performed as a standard protocol to examine the protein expression of PAF1. Slides (4 μm) were prepared from tumor and healthy cervical tissues that were deparaffinized with xylene and dehydrated in alcohol. The slides were incubated overnight with primary antibody (anti-human PAF1, ab137519, Abcam) at 4 °C. The sections were incubated with secondary antibody for 50 min. Representative color images were taken using a microscopic imaging system (NIKON DS-U3, Nikon Corporation, Tokyo, Japan) and examined for positive staining, with brown stained areas indicating positive expression of the PAF1 protein. The mean optical density (MOD) value of the positive stained areas was then measured using Image Pro Plus 6.0 software (Media Cybernetics, MD, USA) with the examiner blinded to the experimental groups.

### Immunofluorescence analysis

The deparaffinization and dehydration of 4 μm tissue slides from tumor and healthy cervical tissues were performed as described above for the IHC procedure. The autofluorescence of the sections was quenched by autofluorescence quenching reagent. After washing in PBS, the tissue sections were blocked with 10% BSA for 30 min and rewashed in PBS. Next, the sections were incubated overnight with the following primary antibodies at 4 °C: anti-PAF1 (sc-514,491, Santa Cruz Biotechnology) and anti-IER5 (NBP1–85935, Novus). The tissue sections were washed in PBS with 0.1% Tween 20 (PBST) 3 times (5 min each time) and then incubated with secondary antibodies, which were fluorescence-tagged with both Texas red and FITC, for 50 min at room temperature and washed again with PBST 3 times. Finally, a drop of anti-fade reagent with DAPI (G1012, Servicebio Technology Co., LTD) was added to the sections, and the coverslips were mounted and examined with a fluorescence microscope (NIKON ECLIPSE C1, Nikon Corporation, Tokyo, Japan).

### Flow cytometry (FCM)

For the FCM this assay, a total of 2.5 × 10^5^ Siha and Hela cells were seeded per well in a six-well plate. The cells were then transfected with a specific siRNA against PAF1 and scrambled RNAi for 48 h and then irradiated. The apoptosis and cell cycle assays were both carried out with these cells after 72 h of radiation intervention. The cells were trypsinized, counted and washed with PBS, and annexin V was used to measure the percentage of apoptotic cells and level propidium iodide staining using an annexin V-FITC/PI apoptosis detection kit (KGA108, KeyGen Biotech, China) followed by FCM (LSR Fortessa SORP, Becton, Dickinson, and Company). The distribution of the cell cycle was measured by flow cytometric analysis (EPICS XL -MCL, Beckman Counter, Inc., CA, USA) of propidium iodide-stained, ethanol-fixed cells using a cell cycle detection kit (KGA512, KeyGen Biotech, China). The data were analyzed using FlowJo X software (FlowJo LLC, OR, USA).

In addition, 2.5 × 10^5^ Siha and Hela cells were seeded per well in a six-well plate for a caspase-3 green flow cytometry assay. The cells were transfected with si-PAF1 or si-IER5, or both, and scrambled RNAi for 48 h, followed by radiation treatment. The caspase-3 green flow cytometry assay was carried out with these cells after 24 h of radiation intervention. A fluorescein active caspase-3 staining kit (Abcam, ab65613) was used to identify cells with active caspase-3, according to the manufacturers’ instructions. The data were acquired using CellQuest 3.2 software (BD Bioscience). Each test was carried out in triplicate. DNA content and cells were analyzed by FCM (LSR Fortessa SORP, Becton, Dickinson, and Company). Each test was conducted three times.

### Proliferation assay

Cell Counting Kit-8 (CCK-8, Solarbio Science & Technology Co., Ltd., Beijing, China) was used to measure cell proliferation according to the manufacturer’s protocol. The assay was performed with 5 × 10^3^ cells seeded in each well of a 96-well plate, under the conditions described above, with four replicate wells in each experimental set. After 48 h of radiation intervention, CCK-8 solution (10 μl) was added to each well, and the cells were incubated at 37 °C for 2 h. Cell proliferation was measured according to the absorbance at 450 nm and compared with to the that of the control (untreated) cells.

### Chromatin immunoprecipitation with quantitative PCR (ChIP-qPCR)

ChIP assays were performed using a SimpleChIP Plus sonication chromatin IP kit (Cell Signaling Technology) according to the manufacturer’s instructions. Antibodies against IgG were used as negative controls, and antibodies against histone H3 were used as positive controls. Siha and Hela cells (10^7^) subjected to IR were fixed with 1% formaldehyde to cross-link DNA and proteins, chromatin was sheared using a Microson Ultrasonic Cell Disruptor XL (Misonix) (10 cycles of sonication: 10 s each, 1-min rest; amplitude = 10, power = 15 W), chromatin (10 μg) was incubated overnight with antibodies (5 μl of PAF1 or Pol II, 10 μl of histone H3-positive control, and 2 μl normal rabbit IgG negative control) at 4 °C, and immunoprecipitates were then bound to protein G magnetic beads (30 μl). The protein-DNA cross-linking was reversed, followed by purification of the DNA, and the enrichment of DNA fragments was determined using qPCR. The qPCR primers used for the ChIP assay are listed in Table S2. The data were normalized and analyzed using fold enrichment analysis as described previously [28]. The promoter-proximal region of IER5 is defined as the region from 200 bp upstream to 300 bp downstream of the TSS and the gene body is defined as the region covering 300 bp to 1.5 kb downstream of the TSS. For the ChIP of Pol II, we conducted qPCR of IER5 promoter and gene body respectively, and obtained the Pol II release ratio (PRR) by calculating the following formula:

$$ PRR=\frac{Gene\ body}{Promoter} $$

### Clustered regularly interspaced short palindromic repeats (CRISPR)/Cas9-mediated IER5 enhancer1/2 knockout

CRISPR/Cas9 single-guide RNA (sgRNA) was used to delete the corresponding genome region containing the two putative enhancers of IER5. The targeted sgRNAs 5′-TCCCGCCTTTCCGGGCCCGG-3′ for upstream and 5′-GTAGGAAATGTTTTCAATGG-3′ for downstream were designed (http://crispr.mit.eud/) and was cloned into lentiCRISPR v2 (Addgene #52961) and co-transfected with packaging plasmid into 293 T cells to produce virus co-expressing sgRNA and Cas9. The virus was then used to infect Siha and Hela cells to produce single-cell clones with homozygous knockouts and the enhancers knockout was analyzed by Sanger sequencing (Fig. S5).

### Statistical analysis

Statistical analysis was performed using SPSS Statistics 25.0 (SPSS, Inc., Chicago, USA) and Prism 7.0 software (GraphPad Software, version 7.0, San Diego, CA, USA). The data are expressed as the mean ± standard error of the mean (SEM) or median (interquartile). Experimental and control groups were compared using two-independent sample t-tests or Mann-Whitney U tests for parametric data and χ^2^ tests and Fisher exact tests for nonparametric data. A value of *p* < 0.05 was considered significant.

## Results

### High expression of PAF1 in human CC samples

**Fig. 1:**
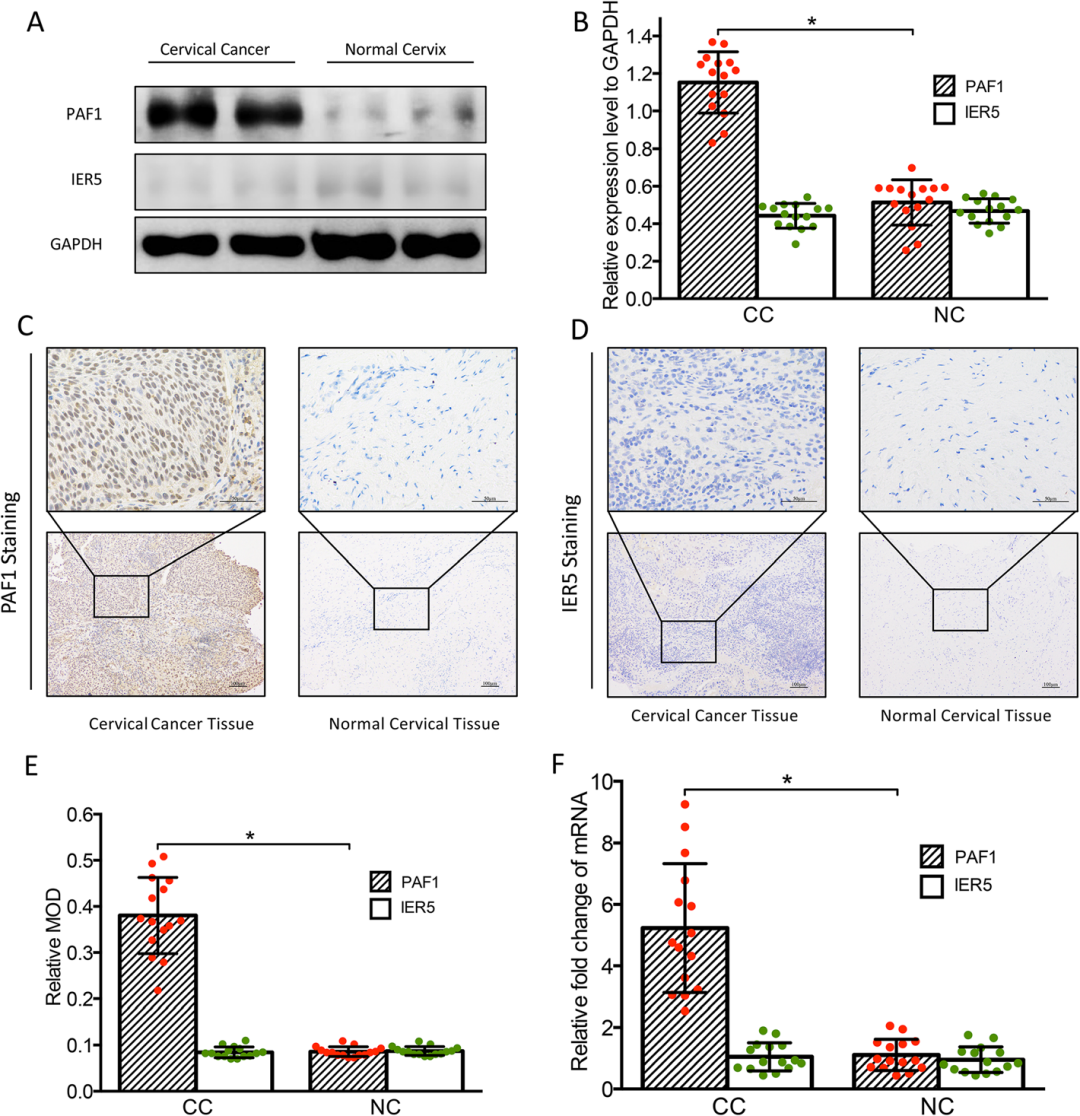
Expression of PAF1 and IER5 in cervical cancer samples. PAF1 and IER5 levels were detected by Western blotting: **(a)** Representative immunoblots showing PAF1 overexpression in CC sample cells and very low expression of IER5 in both cervical cancer and normal cervix; (**b)** Densitometric quantification of PAF1 and IER5 in the CC and NC groups, as evaluated by Western blotting. PAF1 and IER5 levels were also detected by IHC: Representative images of PAF1 **(c)** and IER5 **(d)** proteins staining of cervical cancer and normal cervical tissues by IHC, taken at 100× (scale bar = 100 μm) and 400× (scale bar = 50 μm) magnification; **(e)** Relative MOD quantification of PAF1 and IER5 in the CC and NC groups. **f** Quantitative RT-PCR analysis showed increased expression of PAF1 in the CC sample cells, and low expression of IER5 in both CC and NC groups, normalized to that of β-actin. All data are expressed as the mean (S.D.): * *P* < 0.05, **P < 0.01, *** *P* < 0.001. NC, normal cervix; CC, cervical cancer

Recent studies have reported that PAF1 is an oncogene and is closely related to tumor differentiation, metastasis, therapeutic resistance, and prognosis [20–22]. To investigate the clinical significance of PAF1 in CC pathogenesis, the PAF1 expression pattern was analyzed in healthy and CC tissues. In this study, we detected the expression level of PAF1 and IER5 proteins by western blotting (Fig. 1a, b) and IHC (Fig. 1c, d, and e), both of which showed PAF1 overexpression in CC (*n* = 15) tissue but not in healthy cervical tissue (*n* = 15) while low IER5 expression in CC and healthy cervical tissue. We also measured the mRNA level of PAF1 and confirmed the specific upregulated transcription of PAF1 in CC (Fig. 1f). Overall, these results suggest that PAF1 is overexpressed explicitly in the CC population.

### PAF1 negatively regulates the expression of IER5 in CC cells

**Fig. 2:**
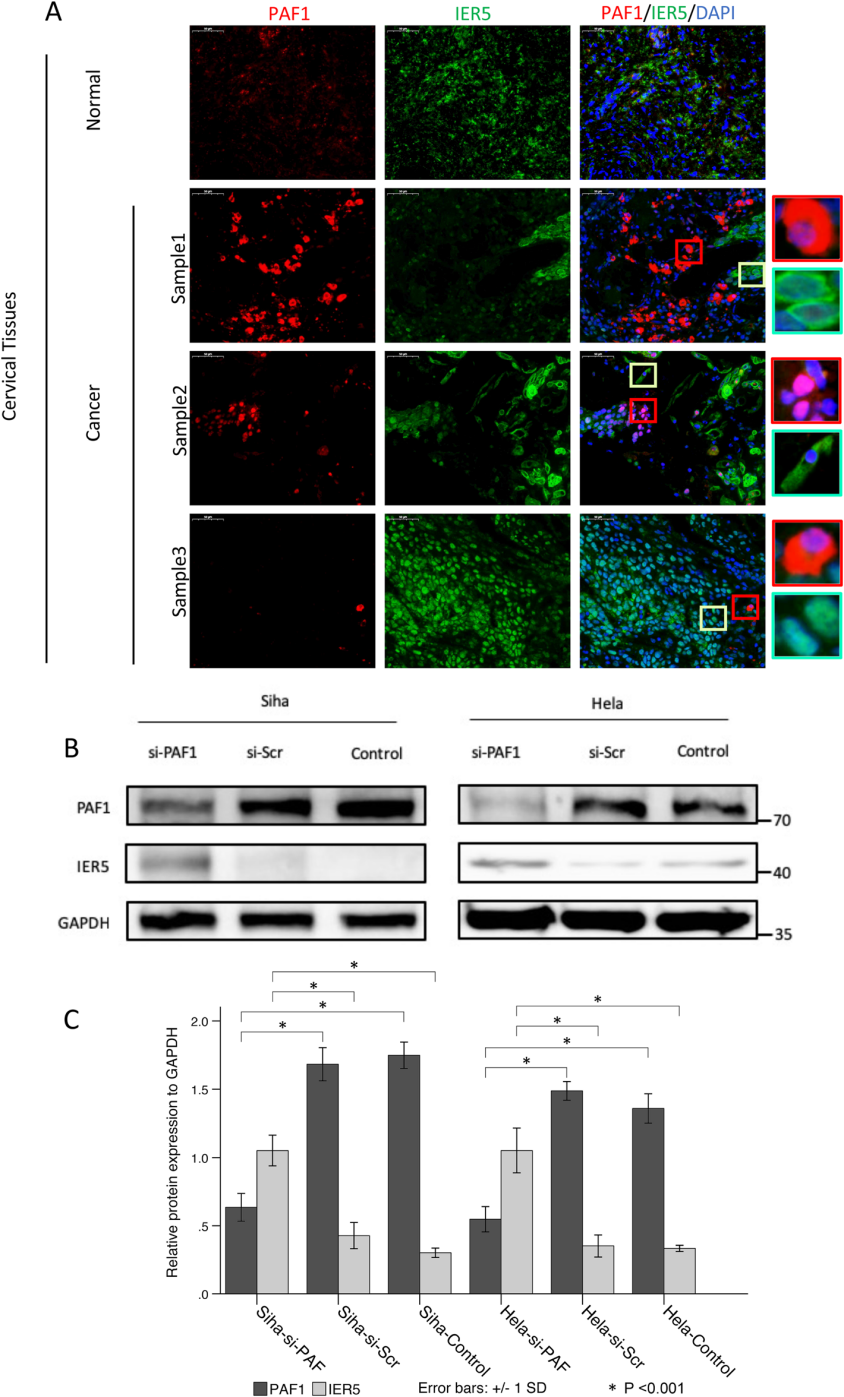
PAF1 regulates the expression of IER5 in CC. **a** Confocal images showing complementary expression of PAF1 and IER5 in CC cells: overexpression of PAF1 in cells with negligible IER5 expression and low levels of PAF1 expression in cells with highly expressed IER5; the highlighted boxes show the magnified images of single-cell staining. The normal control cervical tissue showed no expression of PAF1 or IER5. DAPI was used for nuclear counterstaining. **b, c** Western blotting analysis results showing a decrease in the expression of IER5 protein in the PAF1-knockdown cells. GAPDH was used as an internal control protein. All data are expressed as the mean (S.D.): * *P* < 0.001)

Since PAF1 is highly expressed in CC tissue and, as explained above, PAF1 promotes the release of Pol II on the IER5 promoter in colon cancer cells, we decided to study further the relationship between PAF1 and IER5 in CC cells. First, we determined the localization and expression of both the PAF1 and IER5 proteins in tissues by immunofluorescence confocal microscopy. Interestingly, the results showed that the expression of IER5 was low in cells with high levels of PAF1 expression in CC tissues; conversely, the expression of IER5 was higher in cells with lower PAF1 levels, suggesting a potentially negative regulatory relationship between PAF1 and IER5 in CC cells (Fig. 2a). Furthermore, we transiently knocked down PAF1 using siRNA in both Siha and Hela cells, which resulted in a significant increase in the expression of IER5, demonstrating that PAF1 negatively regulated the IER5 expression in the CC cells (Fig. 2b and c).

### PAF1 attenuates the radiosensitivity of cervical cancer cells by inhibiting IER5

**Fig. 3:**
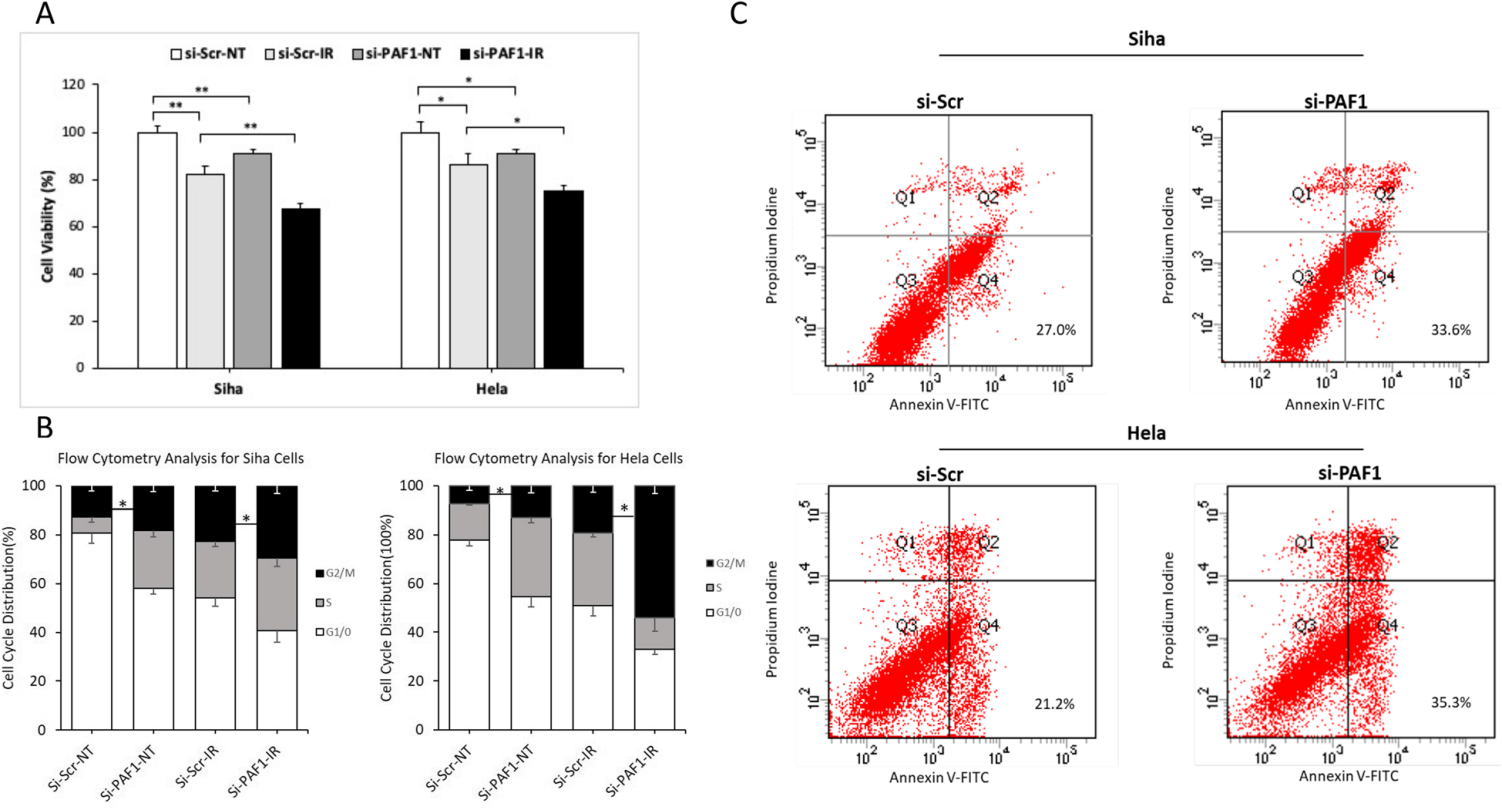
Knocking down PAF1 enhances the efficacy of the radiotherapy of CC cells. **a** CCK-8 assay of Siha and Hela cells treated with radiation showing significantly reduced proliferation rates (Siha cells: *P* = 0.002, Hela cells: *P* = 0.019), and knocking down the PAF1 gene enhanced the cell-killing ability upon cell IR (Siha cells: *P* = 0.004, Hela cells: *P* = 0.018). **b** FCM-cell cycle analysis (24 h after IR) with propidium iodide (PI) staining shows PAF1 knockdown and IR resulted in an increased percentage of both Siha and Hela cells blocked in the G2/M cell cycle. **c** FCM-apoptosis analysis (72 h after cell IR) with PI and annexin V-FITC staining shows an increased percentage of apoptotic PAF1-knockdown Siha and Hela cells (Siha cells: 33.6%, Hela cells: 35.3%) subjected to IR (Siha cells: 3.5 Gy, Hela cells: 4 Gy) compared with that of the control cells (Siha cells: 27.0%, Hela cells: 21.2%), *N* = 3 in each group. All data are expressed as the mean (S.D.): * *P* < 0.05 and ** *P* < 0.01

To investigate the effects of PAF1 on radiosensitivity of CC, we treated Siha and Hela cells with 3.5 Gy radiation followed by transiently knocking down PAF1 using siRNA. Above all, significantly decreased cell proliferation was found by CCK-8 assay (Siha cells: *P* = 0.002, Hela cells: *P* = 0.019) (Fig. 3a), and an increased percentage of cells were found in the G2/M phase (Siha cells: *P* = 0.04, Hela cells: *P* = 0.048) (Fig. 3b) upon radiation treatment. Moreover, PAF1 knockdown in the Siha and Hela cells caused an diminished cell proliferation (Fig. 3a), an increased number of cells arrested in the G2/M phase (Fig. 3b), and increased apoptosis rate (Fig. 3c), after radiation treatment. These results indicate that PAF1 is involved in the control of the radiation resistance process.

**Fig. 4:**
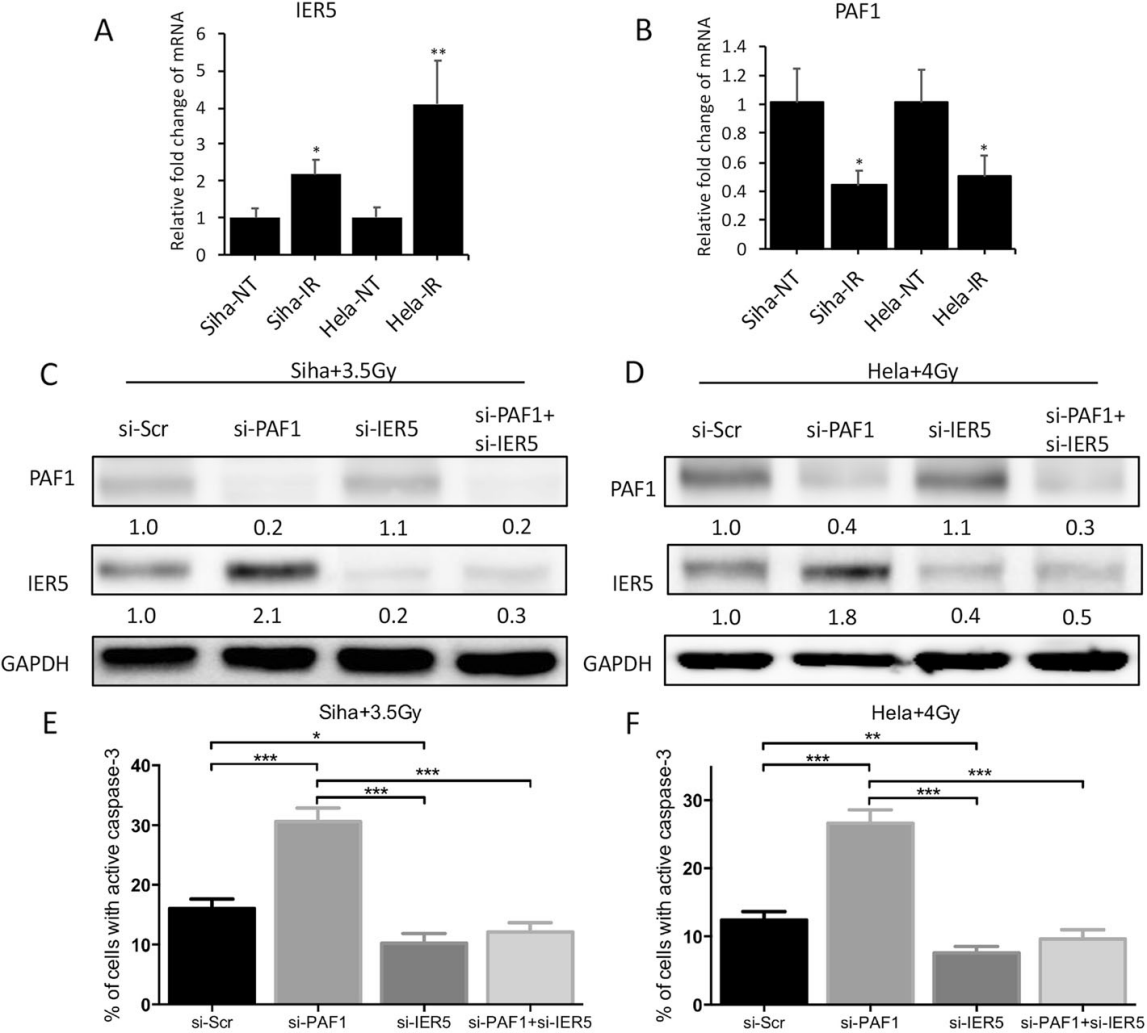
Knocking down PAF1 affects radiosensitivity of CC by regulating IER5. **a**&**b** qRT-PCR assays of mRNA expression in radiation-exposed and unexposed Siha and Hela cells showing increased expression of IER5 and reduced PAF1 after radiation. β-actin served as expression controls. Data are expressed as the mean (S.D.) (*n* = 3). **c**&**d** Western blotting analysis results showing that based on radiation, knocking down PAF1 promoted the expression of IER5, while knocking down IER5 has no significant effect on PAF1. Fold change of intensity is mentioned in the western blotting bands. GAPDH served as expression controls. **e**&**f** FCM assay for analyzing the percentage of cells with active caspase-3 showing that PAF1 knockdown resulted in an increased percentage of cell with active caspase-3 and simultaneous PAF1 and IER5 knockdown resulted in little effect on the apoptosis proportion. *N* = 3 in each group. All data are expressed as the mean (S.D.): * *P* < 0.05 and ** *P* < 0.01

To further verify whether PAF1 affects the radiation sensitivity of CC by regulating IER5, we knocked down the expression of IER5 or PAF1 under radiation treatment, respectively or simultaneously. Firstly, the mRNA level of IER5 was increased but the mRNA level of PAF1 was reduced by radiation (Fig. 4a&b). In the context of radiation, knocking down PAF1 promoted the expression of IER5, while knocking down IER5 has no significant effect on PAF1 (Fig. 4c&d), which suggested that PAF1 regulated the expression of IER5 during radiation. Furthermore, by analyzing the percentage of cells with active caspase-3, which represents the rate of apoptosis, we found that the proportion of apoptotic cells was significantly increased by knocking down PAF1, while the simultaneous knock down of PAF1 and IER5 had little effect on the apoptotic cell proportion (Fig. 4e&f). These results suggest that PAF1 regulates the radiation sensitivity of cervical cancer cells by inhibiting the expression of IER5.

### Binding of PAF1 to the IER5 promoter and enhancers

**Fig. 5:**
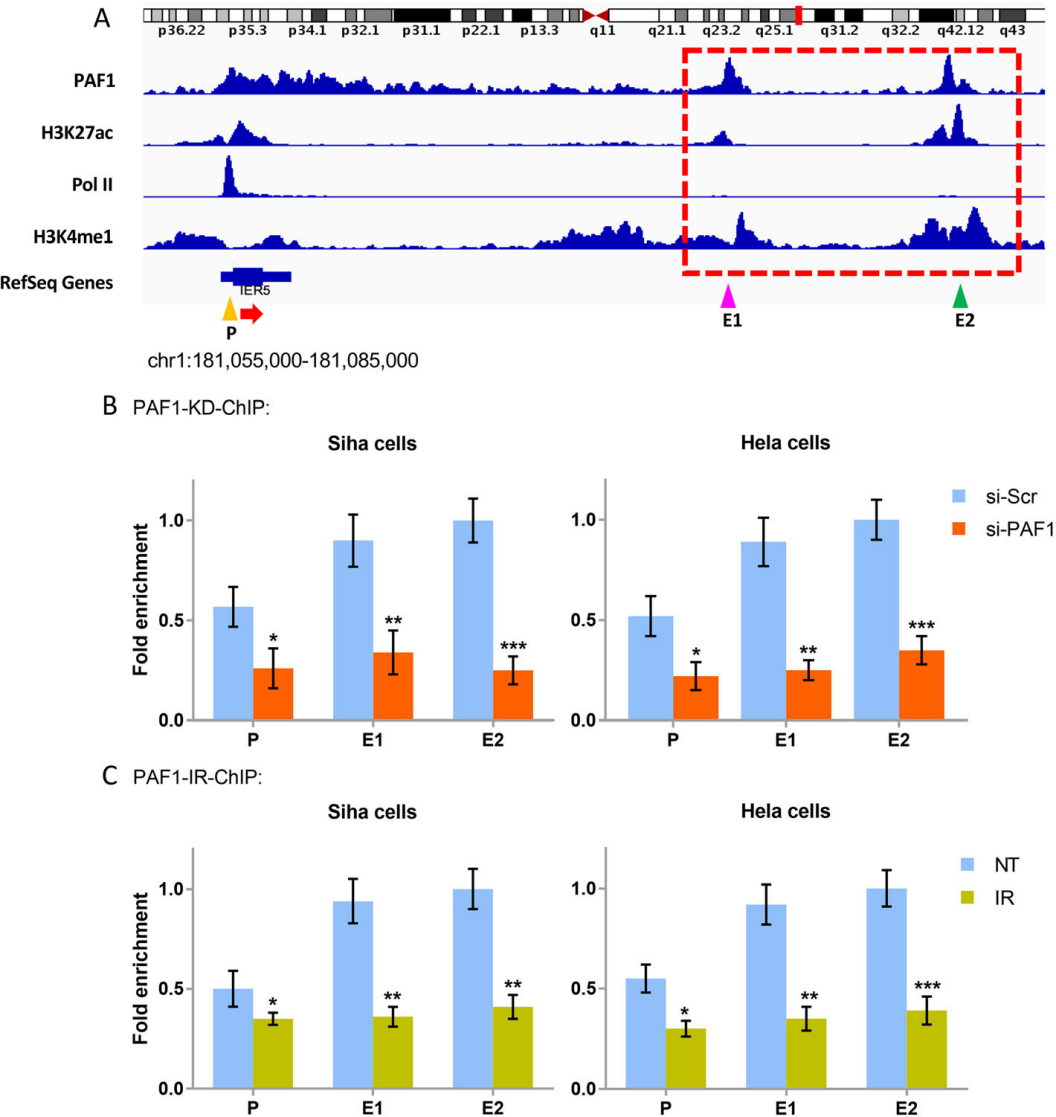
Validation of PAF1 occupancy at the IER5 promoter and enhancers in different situations. **a** Genome browser track examples showing ChIP-seq data of PAF1, H3K27ac, Pol II, and H3K4me1 at the loci of IER5 and the putative enhancer region (red dotted box) aligned to the human genome (GRCh37.p13) using Integrative Genomic Viewer (IGV 2.4.10). The yellow arrow refers to the promoter region (− 199 ~ + 1); the pink arrow refers to the first enhancer region (+ 16,561 ~ + 16,362); the green arrow refers to the second enhancer region (+ 23,662 ~ + 23,861); corresponding primers were designed according to these regions. “+” indicates downstream of the IER5 start codon. P, promoter; E1, enhancer 1; E2, enhancer 2. **b** and **c** ChIP-qPCR results of the occupancy of PAF1 at the two enhancers and the promoter of IER5 in the Siha and Hela cells in different situations. **b** PAF1 occupancy at the promoter and two enhancer regions in the IER5 gene with or without PAF1 depletion in Siha and Hela cells. **c** PAF1 occupancy at the promoter and two enhancer regions of the IER5 gene before or after Siha and Hela cell IR, *N* = 3 in each group. All data are expressed as the mean (S.D.): * *P* < 0.05; ** *P* < 0.01; and *** *P* < 0.001

We analyzed the PAF1 ChIP-Seq data by searching the NCBI SRA SRP103756 database and found that PAF1 has two high-level binding peaks in the putative enhancer region downstream of the IER5 gene, and we also found a PAF1 binding peak in the promoter region of the IER5 gene, but it was not as tall or sharp as that representative of the enhancer region (Fig. 5a). Previously, we found that IER5 is induced by radiotherapy in CC cells and is closely related to apoptosis and cell cycle changes upon radiation exposure [25, 29]. Moreover, the results in this study show that PAF1 plays an essential role in regulating IER5 expression and cell phenotype during CC cells radiation intervention. Thus, we decided to investigate further how PAF regulates the promoter and enhancers of IER5 in different CC cells (Siha and Hela cells) exposed to radiation.

Based on the binding peak of PAF1, we predicted the sequence of DNA to which PAF1 may bind in the promoter and two enhancers regions of IER5, as shown in Fig. S1 to Fig. S4, and the corresponding primers were designed accordingly. By using the ChIP-qPCR approach with a specific anti-PAF1 antibody, we sought to determine whether PAF1 can bind to the promoter or the two enhancers in the IER5 gene. Our results showed that PAF1 had the highest binding affinity for enhancer 2, followed by enhancer 1. Unexpectedly, the binding strength of PAF1 to the promoter region was only approximately one-half that of PAF1 to enhancer 2 (Fig. 5b). After knocking down PAF1 expression, the number of IER5 enhancers enriched with PAF1 was significantly decreased in the precipitation assay (Fig. 5b); interestingly, this phenomenon also occurred after radiation intervention (Fig. 5c). The single most striking observation to emerge from the data above was that PAF1 occupies IER5 enhancers under normal conditions, and the depletion of PAF1 by siRNA or IR causes low binding affinity for these enhancers.

### Effect of PAF1 on pol II pause release at IER5 promoter-proximal region via regulating enhancers

**Fig. 6:**
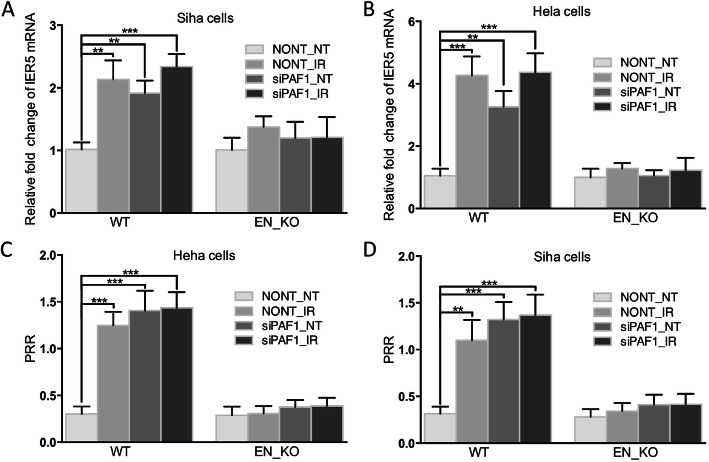
PAF1 promotes Pol II promoter-proximal pausing and causes decreased transcription of IER5 by regulating the enhancers. **a**&**b** Results of qRT-PCR for IER5 mRNA showing that knockout of the enhancers caused PAF1 knockdown or IR to no longer promote IER5 transcription in either Siha (**a**) nor Hela (**b**) cells. **c**&**d** ChIP-qPCR analysis of Pol II occupancy at the promoter and gene body of IER5 revealing that both PAF1 knockdown and IR increased the PRR value, while after knocking out the enhancers, PAF1 knockdown or IR had almost no effect on PRR in Siha (**c**) and Hela (**d**). *N* = 3 in each group. All data are expressed as the mean (S.D.): * *P* < 0.05; ** *P* < 0.01; and *** *P* < 0.001. si-PAF1, PAF1 knockdown; NONT, negative control for PAF1; EN_KO, enhancer1/2 knockout; WT, wild type as negative control for EN_KO; IR, irradiation treatment; NT, non-treatment; PRR, Pol II release ratio

To verify whether the above two enhancers are the main agencies through which PAF1 negatively regulates the transcription of IER5, we knocked these enhancers out by CRISPR/Cas9 and found that the induction effect of PAF1 depletion by siRNA or IR on the transcription of IER5 disappeared (Fig. 6a&b). Turning now to the experimental evidence on Pol II ChIP (Fig. 6c&d), analysis of the relative amount of Pol II in IER5 promoter-proximal region and gene body was performed and the results indicate that depletion of PAF1 increased the Pol II release ratio with or without IR treatment. It is worth noting that after enhancers knockout, the above changes no longer presented. Taken together, these results show that PAF1 promotes Pol II pausing at the IER5 promoter-proximal region and inhibits the transcription of IER5 primarily by regulating the enhancers. This regulation may be part of the mechanism that induces the high expression of the IER5 gene in cells exposed to radiation.

## Discussion

PAF1 is an essential functional subunit of PAF1C and remains highly conserved among different species [17, 30]. Although PAF1 is a subunit of PAF1C, it functions independently in the process of carcinogenic transformation (mostly independently of the other subunits), including tumor cell cycle alterations, chromatin composition changes, tumor cell renewal and tumor stem cell pluripotency [18]. The PAF1 gene is located in the 19q13.2 amplicon locus and was first identified in poorly differentiated pancreatic cancer cell lines (expressed 30-fold greater than that of well-differentiated cancer cell lines) [31]. In the years following its discovery, PAF1 has been found to be highly expressed in various cancers and is associated with tumor metastasis, diminished differentiation, therapeutic sensitivity, and poor prognosis [20, 21]. In the current study, we demonstrated, for the first time, that the expression of PAF1 was significantly enriched in CC cells. Moreover, the expression level of PAF1 in both the Siha and Hela cells was decreased after radiation treatment. These results suggest that PAF1 is involved in the development of CC and its response to radiation treatment.

Studies in recent years have suggested that PAF1 is a pivotal transcription factor for certain “induced” genes [32]. Since the results from our earlier studies demonstrated that the expression of IER5 could be induced in radiation-exposed CC cells [12], in this study, we correlated PAF1 with IER5 in both CC tissue and different cell lines. First, the localization analysis of IER5 in cells from CC tissue revealed anti-correlated expression with PAF1. In addition, the analysis of IER5 and the cell phenotypes after PAF1 knockdown showed an increased level of expression in the si-PAF1 group along with lower levels of cell proliferation and an increase in G2/M cell cycle arrest compared with these measures in the control cells. These findings, in parallel with the complementary locational expression of IER5 and PAF1, prompted us to propose that PAF1 may be involved in the IER5 upregulation induced by the radiation treatment of CC cells.

Our previous studies showed not only that IER5 is induced by IR but also that IER5 is involved in the regulation of radiosensitivity in CC cells by promoting their apoptosis [13, 14]. In this study, we found, after cell treatment with radiation, that the expression of PAF1 was decreased and that IER5 was upregulated, and PAF1 knockdown and radiation treatment together significantly increased IER5 expression and led to an obvious increase in the apoptotic cells; synergistically, more cells were arrested in the G2/M phase, while simultaneous PAF1 and IER5 knockdown had little effect on the proportion of apoptotic cells. Taken together, these results indicate that PAF1 affects the radiation sensitivity of CC cells by regulating IER5 expression.

A recent report showed that the negative transcriptional regulation of the IER5 gene at its promoter region was abolished, which resulted in increased IER5 transcription upon the IR of HepG2 cells [15]. With the development of tumor genomics, an increased number of activated enhancers have been found in many malignant tumors, promoting gene transcription exponentially in a variety of ways [33]. Coincidentally, such activated enhancers have been found near the IER5 gene locus and rapidly promote transcription of the IER5 gene under the induction of external stimuli in several solid tumors, including CC [16]. Furthermore, it was demonstrated that, when DNA is severely damaged by radiation, the IER5 enhancer is activated, which promotes the binding of Pol II to the promoter and induces the transcription of the IER5 gene [16]. These studies suggested that the main reason for the upregulation of radiation-induced IER5 may be the increased transcription caused by the enhancer.

Since PAF1 is a major transcription factor and can negatively regulate the expression of IER5 in CC cells, understanding the mechanism by which PAF1 affects the expression of IER5 through promoters and enhancers was expected to provide considerable insight into the radiation resistance of CC. In this context, we predicted the promoter and two presumptive enhancers for the IER5 gene, illustrating, for the first time, that the relative occupancies of PAF1 are much higher on the two enhancers than on the promoter, and this interaction of PAF1 with the IER5 enhancers was reduced by PAF1 knockdown, indicating a notable role for the diminished enhancer activity induced by PAF1. Interestingly, the binding of PAF1 to the two enhancers of IER5 was weakened after cell IR, which may have been due to the decreased expression of PAF1 upon IR. Our results also showed that these two enhancers are the main agencies through which PAF1 negatively regulates the transcription of IER5. As a Pol II-related factor, PAF1 is involved in the pausing and releasing of the Pol II at the promoter-proximal region, affecting the transcription initiation and extension for target genes [34]. This study found that the binding of PAF1 to the IER5 enhancers induces paused Pol II at the promoter-proximal region in CC cells exposed to radiation. Therefore, we speculate that the radiation-induced IER5 expression is likely due to the promoted Pol II release and the upregulated transcription caused by abolishing the inhibitory role of PAF1 on the IER5 enhancers.

Further research is needed to verify the main pathway by which PAF1 affects target enhancers. A recent study has shown that PAF1 inhibits the full activation of less-active enhancers; furthermore, the transcription of the enhancer and the occupancy of Pol II on the enhancer were both increased after the inhibition of PAF1 [23]. Therefore, we speculated that PAF1, as a Pol II-associated factor, indirectly regulates the transcription of these enhancers by binding to Pol II, thereby inhibiting the synthesis of eRNA and weakening the long-acting trans-promoting effect of eRNA on the target gene promoter. Considering that eRNA has a low abundance value, the value of the trans-promoting effect of eRNA is debated and needs further study [33]. More importantly, the study also found that the relative occupancy level of PAF1 at the enhancer was much higher than that of Pol II, suggesting that the regulation of PAF1-induced enhancer activity is likely mediated by a mechanism that does not involve Pol II. By analyzing the functional domain of the PAF1 protein, we found that PAF1 has possible DNA-binding domains (HLH and LZ domains); therefore, we speculated that PAF1 likely plays a regulatory role mainly by directly binding the IER5 enhancer.

In conclusion, PAF1 is involved in tumorigenesis and radiosensitivity in CC tissues; the inhibition of PAF1 leads to an upregulated IER5 and increased radiosensitivity. Most importantly, we found that PAF1 occupies two putative enhancers downstream of the IER5 gene and consequently hinders the transcription of IER5 by promoting Pol II pausing at the promoter-proximal region, revealing a layer of IER5 regulation that directly connects PAF1 with enhancer function in response to radiation treatment of CC. Overall, our study suggests that PAF1 may play an essential role in the radiosensitivity of CC, and further investigation about the mechanism of IER5 enhancer regulation, or that of other target genes, by PAF1 is expected to provide critical information for advancing towards the development of novel therapeutic targets to reduce the incidence of radiation resistance in CC patients.

## Supplementary information

**Additional file 1: Figure S1.** Predicted binding peaks of PAF1 on the IER5 gene promoter. The 5′ upstream region of the IER5 gene nucleotide sequence is shown (chr1: 181, 057, 440–181, 058, 239). The PAF1 binding peak is highlighted in red (chr1: 181, 057, 840–181, 058, 039). The position of the peak site relative to TSS (+ 1) is shown.

**Additional file 2: Figure S2.** Predicted binding peaks of PAF1 on IER5 gene enhancer 1. The nucleotide sequence for part of the putative enhancer 1 region in the IER5 gene is shown (chr1: 181, 074, 300–181, 074, 899). The PAF1 binding peak is highlighted in red (chr1: 181, 074, 400–181, 074, 599). The position of the peak site relative to TSS (+ 1) of the IER5 gene is shown.

**Additional file 3: Figure S3.** Predicted binding peaks of PAF1 on IER5 gene enhancer 2. The nucleotide sequence for part of the putative enhancer 2 region in the IER5 gene is shown (chr1: 181, 081, 600–181, 082, 049). The PAF1 binding peak is highlighted in red (chr1: 181, 081, 700–181, 081, 899). The position of the peak site relative to TSS (+ 1) of the IER5 gene is shown.

**Additional file 4: Figure S4.** Predicted negative control region on chr1. The nucleotide sequence of part of the putative negative control region on chr1 is shown (chr1: 181974200–181,975,000). PAF1 has no binding peak for the DNA in this region. The position of the peak site relative to TSS (+ 1) of the IER5 gene is shown.

**Additional file 5: Figure S5.** Deletion of IER5 enhancer1/2 using CRISPR/Cas9 in Siha and Hela cells. Genomic sequences validation of enhancer1/2 knockout by amplifying and Sanger sequencing. Sequences including the putative enhancer 1 and enhancer 2 region of IER5 gene nucleotide sequence was shown (chr1:181,074,364-181,081,980). SgRNA1 for upstream was highlighted in yellow color; sgRNA2 for downstream was highlighted in cyan color; the knockout region was highlighted in red color.

**Additional file 6: Table S1.** List of human qRT-PCR primers used in this study.

**Additional file 7: Table S2.** Related to Supplementary Material and Methods: List of ChIP primers used in this study.

## Data Availability

The data sets used and/or analyzed in this study are available from the corresponding author on reasonable request.

## Abbreviations

CC: Cervical cancer
IER5: Immediate-early response 5
Pol II: RNA Polymerase II
hPAF1: Human RNA polymerase II-associated factor 1
PAF1C: RNA polymerase II-associated factor complex
IR: Irradiation
PBS: Phosphate-buffered saline
RIPA: Radioimmunoprecipitation assay
PMSF: Phenylmethanesulfonyl fluoride
BCA: Bicinchoninic acid
qRT-PCR: Quantitative reverse transcription-polymerase chain reaction
IHC: Immunohistochemistry
PBST: Hosphate-buffered saline with 0.1% Tween 20
FCM: Flow cytometry
CCK-8: Cell Counting Kit-8
ChIP-qPCR: Chromatin immunoprecipitation with quantitative PCR
PRR: Pol II release ratio
CRISPR/Cas9: Clustered Regularly Interspaced Short Palindromic Repeats/Cas9
SEM: Standard error of the mean
