# Supplementary figures and images for: Novel role of PAF1 in attenuating radiosensitivity in cervical cancer by inhibiting IER5 transcription
Source: Radiat Oncol. 2020 May 29;15:131. doi: 10.1186/s13014-020-01580-w (PMC7257241; doi:10.1186/s13014-020-01580-w)

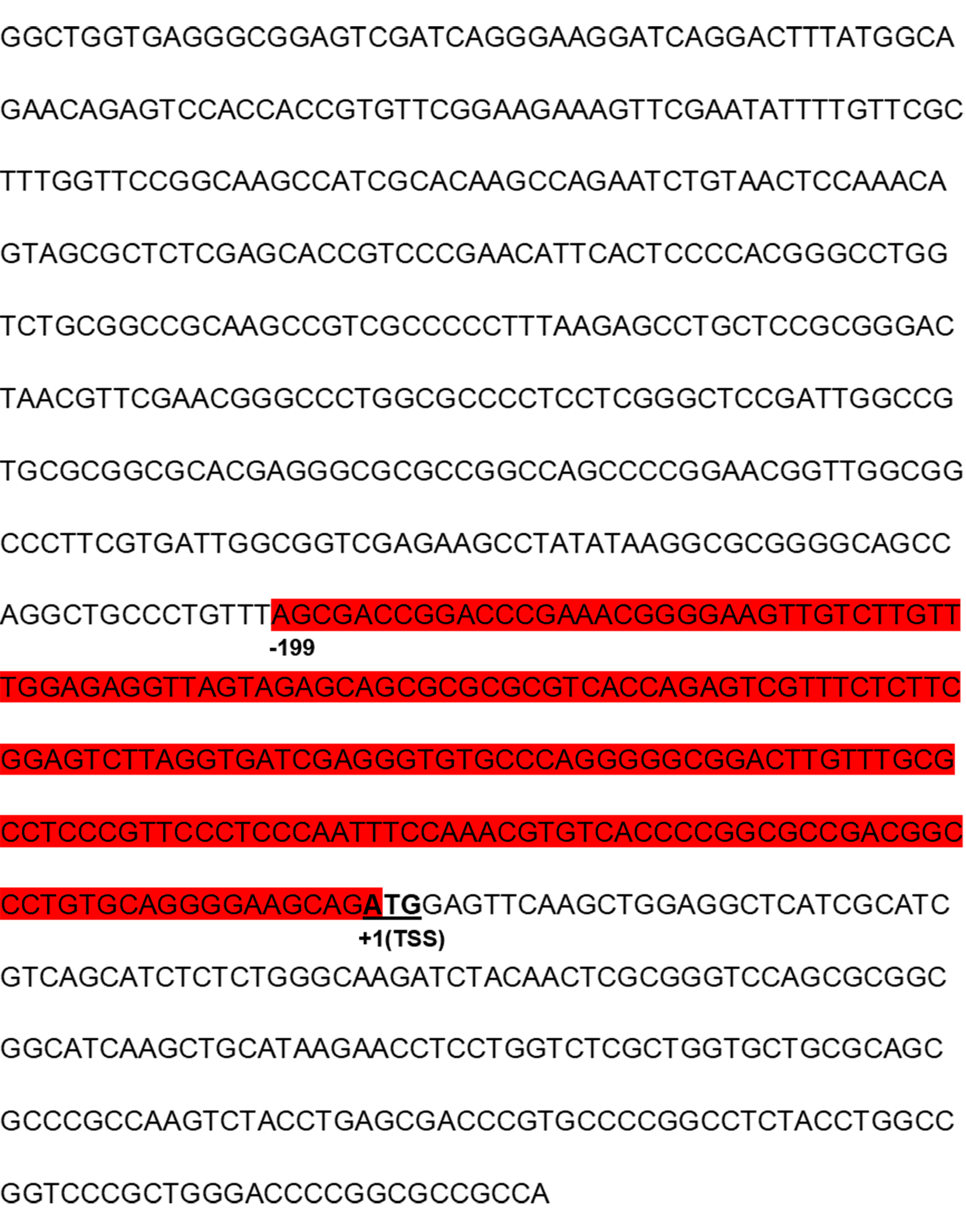

Supplement: Supplementary file 1 — Additional file 1: Figure S1. Predicted binding peaks of PAF1 on the IER5 gene promoter. The 5′ upstream region of the IER5 gene nucleotide sequence is shown (chr1: 181, 057, 440–181, 058, 239). The PAF1 binding peak is highlighted in red (chr1: 181, 057, 840–181, 058, 039). The position of the peak site relative to TSS (+ 1) is shown. [file 13014_2020_1580_MOESM1_ESM.tif]

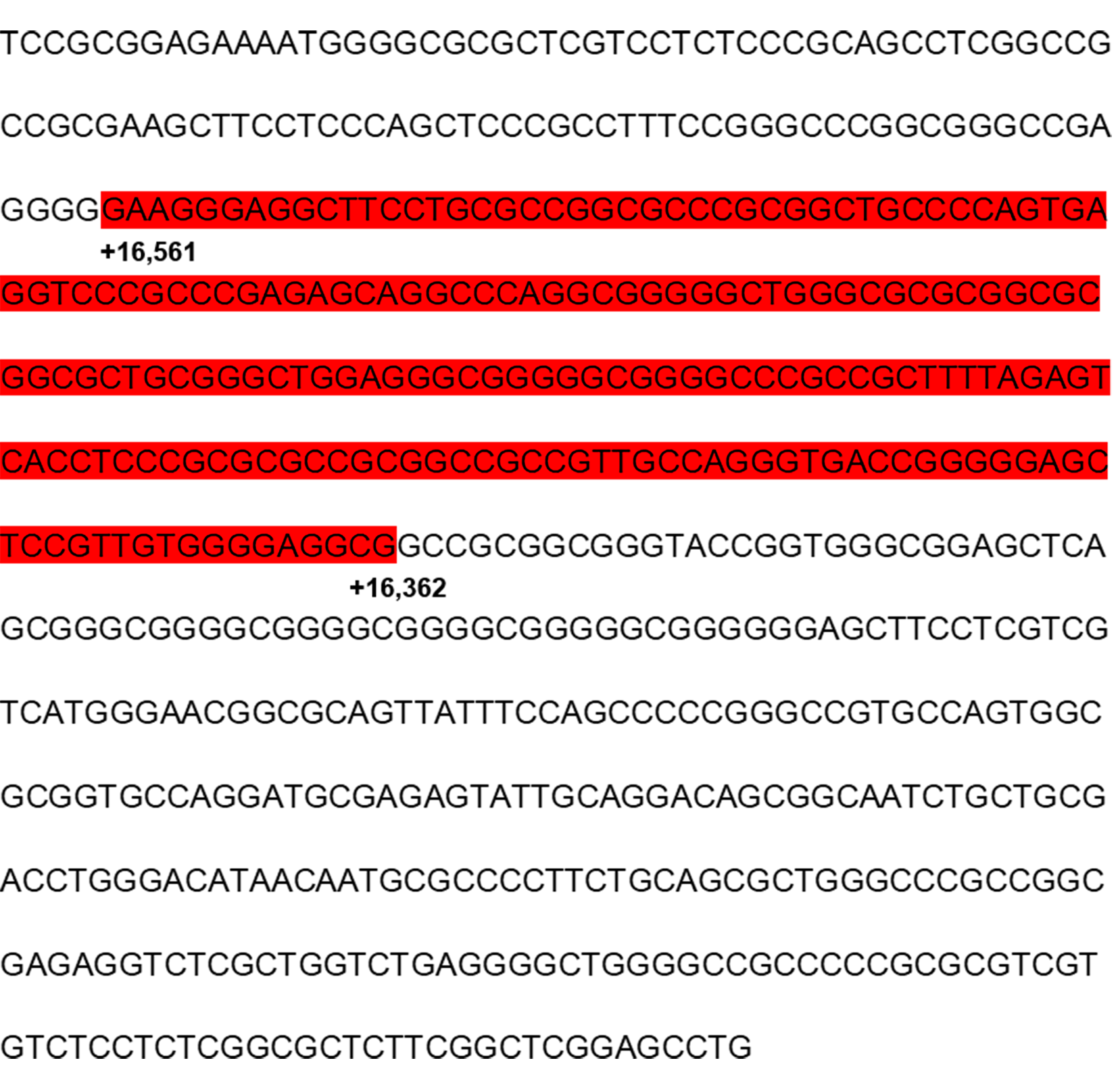

Supplement: Supplementary file 2 — Additional file 2: Figure S2. Predicted binding peaks of PAF1 on IER5 gene enhancer 1. The nucleotide sequence for part of the putative enhancer 1 region in the IER5 gene is shown (chr1: 181, 074, 300–181, 074, 899). The PAF1 binding peak is highlighted in red (chr1: 181, 074, 400–181, 074, 599). The position of the peak site relative to TSS (+ 1) of the IER5 gene is shown. [file 13014_2020_1580_MOESM2_ESM.tif]

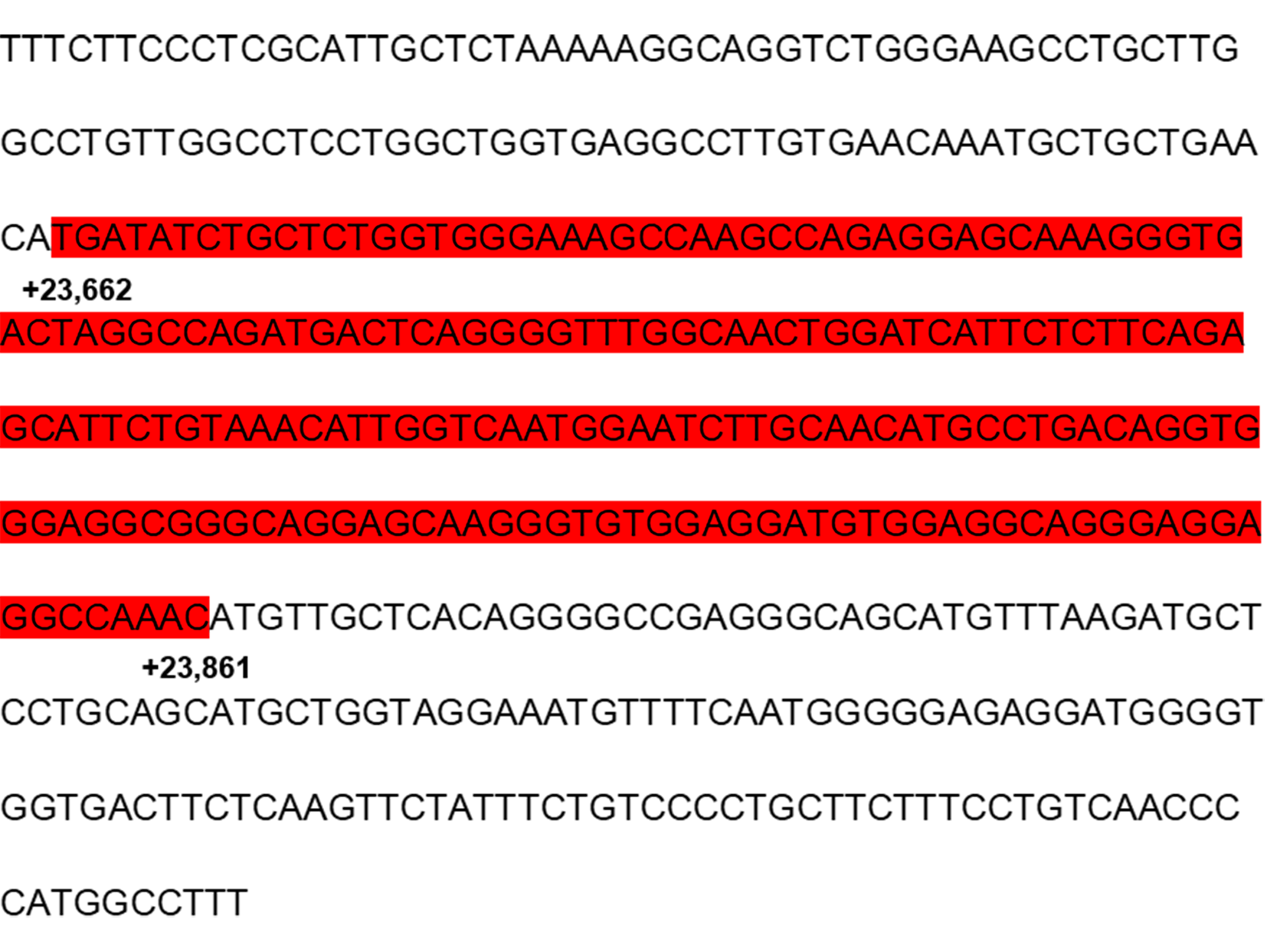

Supplement: Supplementary file 3 — Additional file 3: Figure S3. Predicted binding peaks of PAF1 on IER5 gene enhancer 2. The nucleotide sequence for part of the putative enhancer 2 region in the IER5 gene is shown (chr1: 181, 081, 600–181, 082, 049). The PAF1 binding peak is highlighted in red (chr1: 181, 081, 700–181, 081, 899). The position of the peak site relative to TSS (+ 1) of the IER5 gene is shown. [file 13014_2020_1580_MOESM3_ESM.tif]

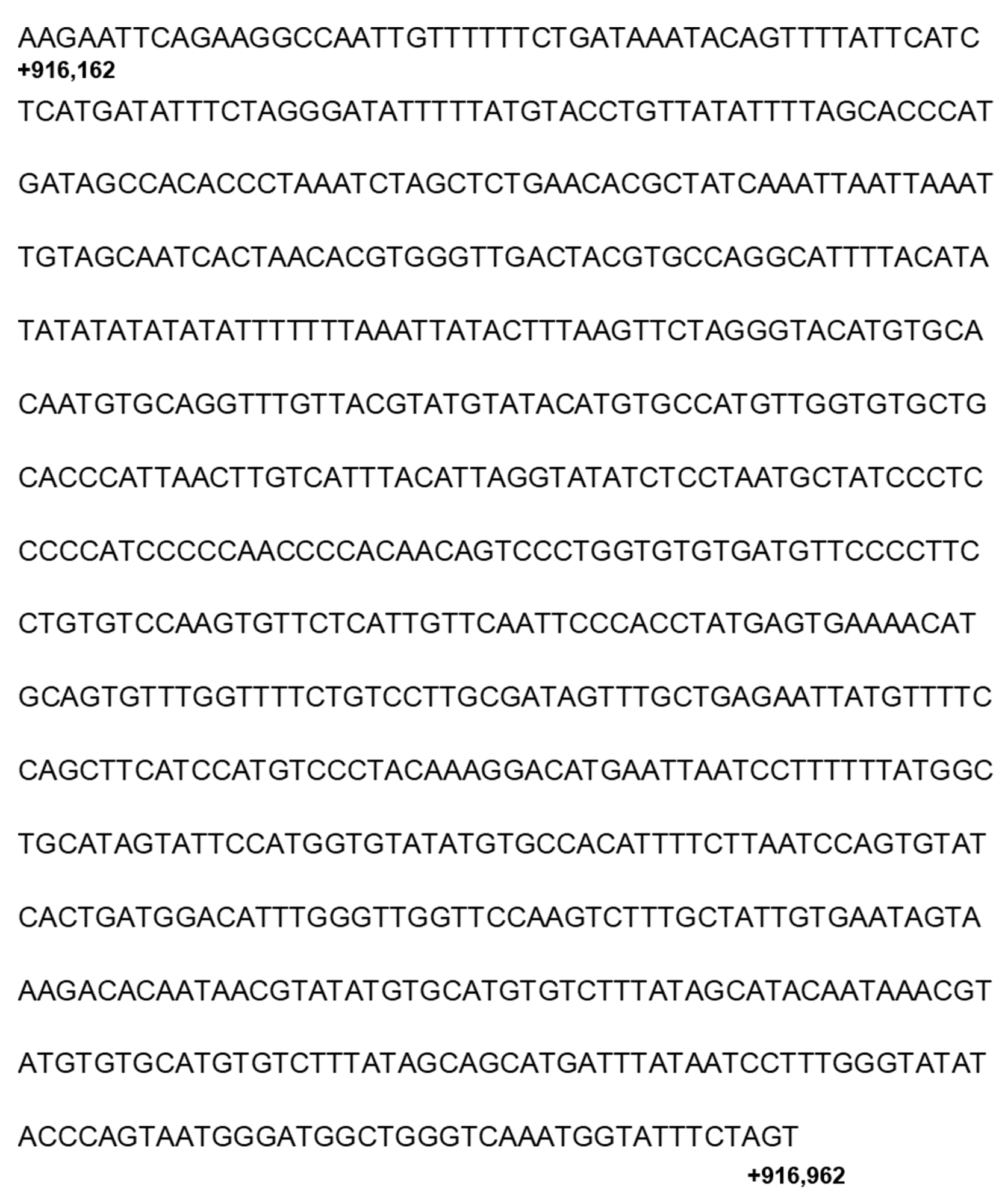

Supplement: Supplementary file 4 — Additional file 4: Figure S4. Predicted negative control region on chr1. The nucleotide sequence of part of the putative negative control region on chr1 is shown (chr1: 181974200–181,975,000). PAF1 has no binding peak for the DNA in this region. The position of the peak site relative to TSS (+ 1) of the IER5 gene is shown. [file 13014_2020_1580_MOESM4_ESM.tif]

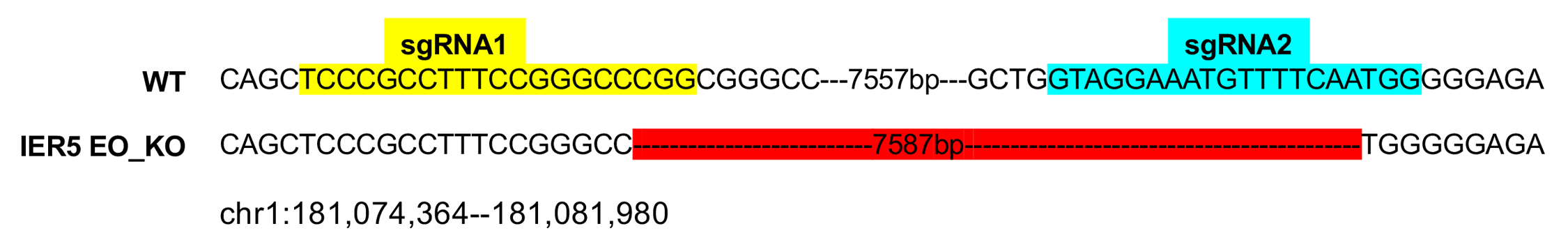

Supplement: Supplementary file 5 — Additional file 5: Figure S5. Deletion of IER5 enhancer1/2 using CRISPR/Cas9 in Siha and Hela cells. Genomic sequences validation of enhancer1/2 knockout by amplifying and Sanger sequencing. Sequences including the putative enhancer 1 and enhancer 2 region of IER5 gene nucleotide sequence was shown (chr1:181,074,364-181,081,980). SgRNA1 for upstream was highlighted in yellow color; sgRNA2 for downstream was highlighted in cyan color; the knockout region was highlighted in red color. [file 13014_2020_1580_MOESM5_ESM.tif]
