# Supplementary material for: Novel role of PAF1 in attenuating radiosensitivity in cervical cancer by inhibiting IER5 transcription
Source: Radiat Oncol. 2020 May 29;15:131. doi: 10.1186/s13014-020-01580-w (PMC7257241; doi:10.1186/s13014-020-01580-w)
Supplement: Supplementary file 6 — Additional file 6: Table S1. List of human qRT-PCR primers used in this study. [file 13014_2020_1580_MOESM6_ESM.docx]

| **Gene Name** | **Forward and Reverse Primers** | **Amplicon Size** |
| --- | --- | --- |
| **PAF1** | F: 5’- CTCACAGCATTACAGCAAACC-3’ | 231 |
|  | R: 5’- GTCTCTTCTACAGGCAGGAAAT-3’ |  |
| **IER5** | F: 5’-GGACGACACCGACGAGGAG-3’ | 183 |
|  | R: 5'- GCTTTTCCGTAGGAGTCCCG-3’ |  |
| **β-actin** | F: 5'-GGACATCCGCAAAGACCTGTA-3' | 143 |
|  | R: 5'-GCTCAGGAGGAGCAATGATCT-3' |  |
