# Supplementary material for: Novel role of PAF1 in attenuating radiosensitivity in cervical cancer by inhibiting IER5 transcription
Source: Radiat Oncol. 2020 May 29;15:131. doi: 10.1186/s13014-020-01580-w (PMC7257241; doi:10.1186/s13014-020-01580-w)
Supplement: Supplementary file 7 — Additional file 7: Table S2. Related to Supplementary Material and Methods: List of ChIP primers used in this study. [file 13014_2020_1580_MOESM7_ESM.docx]

| **Binding Site** | **Forward and Reverse Primers** | **Amplicon Size** |
| --- | --- | --- |
| **Promoter (P)** | F: 5’-ACGGGGAAGTTGTCTTGTTTG-3’ | 121 |
|  | R: 5’-GAAATTGGGAGGGAACGGGAG-3’ |  |
| **Gene body** | F: 5’-TGGAATGCACCACTCCTTCC-3’ | 211 |
|  | R: 5’-GGAAATCCTGTGCGGAAACG-3’ |  |
| **Enhancer 1 (E1)** | F: CAGTTATTTCCAGCCCCCG | 107 |
|  | R: AGGGGCGCATTGTTATGTCC |  |
| **Enhancer 2 (E2)** | F: 5’-ACTCAGGGGTTTGGCAACTG-3’ | 119 |
|  | R: 5’-ACATCCTCCACACCCTTGCT-3’. |  |
| **Negative Control** | F: 5’-TTAGCACCCATGATAGCCACAC-3’ | 100 |
|  | R: 5’-CTGGCACGTAGTCAACCCA-3’ |  |
